# Strange Bedfellows: Nuclear Factor, Erythroid 2-Like 2 (Nrf2) and Hypoxia-Inducible Factor 1 (HIF-1) in Tumor Hypoxia

**DOI:** 10.3390/antiox6020027

**Published:** 2017-04-06

**Authors:** Rachel K. Toth, Noel A. Warfel

**Affiliations:** 1 University of Arizona Cancer Center, Tucson, AZ 85724, USA; racheltoth@email.arizona.edu; 2 Cellular and Molecular Medicine, University of Arizona, Tucson, AZ 85724, USA

**Keywords:** Nuclear factor, erythroid 2-like 2 (Nrf2), hypoxia, reactive oxygen species, hypoxia-inducible factor 1 (HIF-1) signaling, chemoresistance

## Abstract

The importance of the tumor microenvironment for cancer progression and therapeutic resistance is an emerging focus of cancer biology. Hypoxia, or low oxygen, is a hallmark of solid tumors that promotes metastasis and represents a significant obstacle to successful cancer therapy. In response to hypoxia, cancer cells activate a transcriptional program that allows them to survive and thrive in this harsh microenvironment. Hypoxia-inducible factor 1 (HIF-1) is considered the main effector of the cellular response to hypoxia, stimulating the transcription of genes involved in promoting angiogenesis and altering cellular metabolism. However, growing evidence suggests that the cellular response to hypoxia is much more complex, involving coordinated signaling through stress response pathways. One key signaling molecule that is activated in response to hypoxia is nuclear factor, erythroid 2 like-2 (Nrf2). Nrf2 is a transcription factor that controls the expression of antioxidant-response genes, allowing the cell to regulate reactive oxygen species. Nrf2 is also activated in various cancer types due to genetic and epigenetic alterations, and is associated with poor survival and resistance to therapy. Emerging evidence suggests that coordinated signaling through Nrf2 and HIF-1 is critical for tumor survival and progression. In this review, we discuss the distinct and overlapping roles of HIF-1 and Nrf2 in the cellular response to hypoxia, with a focus on how targeting Nrf2 could provide novel chemotherapeutic modalities for treating solid tumors.

## 1. Introduction

Cells and tissue require a delicate balance of many factors to maintain homeostasis in response to external signals. One such signal that is prevalent in disease is lack of oxygen, or hypoxia. Human tissues experience hypoxia owing to various physiological ailments, including sepsis [1], sleep apnea [2], chronic obstructive pulmonary disease [3], diabetic kidney disease [4], and wound healing [5]. However, hypoxia has arguably been most well studied in the context of the solid tumor microenvironment, where it is associated with increased metastasis, resistance to anticancer chemotherapies, and poor patient prognosis. A hallmark of hypoxia is the formation of radical oxygen species (ROS). Although it is not entirely clear why ROS accumulate in hypoxic cells, it is well established that a lack of molecular oxygen shuts down the mitochondrial electron transport chain, which blocks the reduction of oxygen to water coupled with the formation of ATP [6]. Electrons that would normally be funneled into this system are then transferred to oxygen in an incomplete reduction reaction, causing the formation of oxygen radicals, such as superoxide and hydroxyl radicals. These ROS can wreak havoc on various cellular processes, including oxidizing nucleotide bases in DNA and RNA and oxidizing reactive cysteine residues [7]. In addition, ROS can serve as signaling molecules, regulating the activation state of pro-survival pathways that are common drivers of cancer, including phosphatidylinositol-3 kinase and mitogen-activated protein kinase (MAPK) signaling [7]. Thus, strict regulation of ROS in cells is vital for maintaining cellular homeostasis. ROS also play a role in the life cycle of pluripotent and adult stem cells. As with differentiated cells, high ROS levels can negatively impact stem cell homeostasis and may result in apoptosis [8]. However, ROS can also act in signaling to regulate stem cell maturation. Increased levels of ROS in stem cells can promote differentiation over proliferation/renewal [9], and it has been shown that differentiating stem cells actively downregulate expression of the antioxidant regulator nuclear factor, erythroid 2-like 2 (NFE2L2; Nrf2) [10], suggesting that an increase in ROS is necessary for terminal differentiation. Cancer stem cells may take advantage of these systems by depleting ROS, which not only increases survival but also allows them to facilitate migration.

Nrf2 is an important transcription factor that is responsible for maintaining oxidative homeostasis. The expression of Nrf2 is primarily regulated at the post-translational level via proteasomal degradation. In the absence of oxidative stress, Nrf2 is bound by its negative regulator, KEAP1 (Kelch-like erythroid cell-derived protein with cap’n’collar [CNC] homology-associated protein 1). Binding to KEAP1 promotes the ubiquitination of Nrf2 by Cullin3 and facilitates its subsequent degradation by the 26S proteasome [11]. This interaction is abrogated by the presence of oxidative stress, as KEAP1 is oxidized on several key cysteine residues, which alters its conformation and allows Nrf2 to escape ubiquitination-mediated degradation and translocate into the nucleus, where it binds to promoters containing an antioxidant response element (ARE). Activation of Nrf2 promotes the transcription of antioxidant genes, such as heme oxygenase 1 (HO-1) and NADPH (nicotinamide adenine dinucleotide phosphate):quinone dehydrogenase 1 (NQO1) [12], which reduce the levels of damaging ROS in the cell, thus maintaining homeostasis. Regulation of the Nrf2–ARE signaling pathway has been extensively reviewed elsewhere [13,14].

## 2. The Pro-Carcinogenic Role of Nrf2

Owing to its ability to reduce cellular ROS, initial efforts to manipulate Nrf2 in disease focused on its cytoprotective role, with the hypothesis being that increasing Nrf2 activity would be beneficial in preventing and treating certain disease states by decreasing ROS. Indeed, the identification of compounds that activate Nrf2 has been a major focus in drug discovery [15]. However, recent evidence indicates that activation of Nrf2 signaling can also be detrimental, particularly in the context of cancer. Increased Nrf2 expression is correlated with decreased survival and increased metastasis in several cancer types, including gastric cancer, glioma, hepatocellular carcinoma, breast cancer, prostate cancer, and small cell and non-small cell lung cancer [16,17,18,19,20]. Activating mutations in Nrf2, inactivating mutations in KEAP1, and epigenetic dysregulation of *KEAP1* and *NFE2L2* have been observed in various types of cancers [21,22] (Table 1), and these changes are more common in cells and tissues that demonstrate de novo or acquired resistance to various types of chemotherapy [20,23]. Thus, inhibiting the Nrf2–ARE signaling pathway has the potential to improve the efficacy of anticancer therapies and patient prognosis.

In addition to its role in promoting tumor cell survival, Nrf2 can also impact the formation of the tumor vasculature. In glioblastoma samples, Nrf2 expression was correlated with higher microvessel density, and knockdown of Nrf2 reduced endothelial tube formation and blocked vascular endothelial growth factor (VEGF) secretion [34]. In xenograft models of colon cancer, tumors formed by cells with stable knockdown of Nrf2 developed fewer blood vessels, and this was related to reduced expression of hypoxia inducible factor (HIF)-1α [35]. Kozakowska et al. found that patients with bladder cancer who had higher serum levels of Nrf2 and its downstream target, HO-1, also had higher serum and tumor levels of VEGF than patients with lower levels of serum Nrf2. They speculated that Nrf2 may regulate the expression of several pro-angiogenic microRNAs [36].

Nrf2 also contributes to several of the steps required for tumor metastasis. First, multiple studies have shown that increased expression of Nrf2 causes tumor cells to adopt a spindle-shaped morphology, a change indicative of epithelial to mesenchymal transition (EMT) [37]. Activation of Nrf2 signaling also decreased cell–cell contacts in pancreatic duct epithelial cells [37]. In addition, activation of the Janus kinase/signal transducer and activator of transcription (STAT) pathway, which is a known inducer of migration and invasion in vitro, increased Nrf2 activation in pancreatic stellate cells [38]. The observed increase in Nrf2 activation was necessary for the expression of EMT markers, and it was sufficient to increase migration and invasion, even when interleukin (IL)-6 was blocked. Nrf2 also positively regulates Ras family member homolog A, an essential factor that promotes reorganization of the actin cytoskeleton and regulates cell invasion and motility. In breast cancer cell lines, irrespective of hormone receptor status, knockdown of Nrf2 decreased transwell migration and invasion in wound healing assays [39].

One of the best studied Nrf2 target genes is HO-1, a member of the heme oxygenase family, which catalyzes heme degradation. Numerous transcription factors can activate HO-1 expression in response to growth factors and cytokines, including activator protein-1 and nuclear factor κB (NF-κB) [40]. Under normal physiological conditions, the Bach1 (Broad-Complex, Tramtrack, and Bric a brac domain and CNC homolog 1) transcription factor binds small MAF proteins (sMAFs) that recognize Maf-recognition elements in the HO-1 promoter, repressing HO-1 transcription. In cancer cells, transcriptional activators, such as Nrf2 and HIF-1, are expressed at higher levels and outcompete Bach1 for sMAF binding, resulting in the transcription of HO-1 [41,42]. Increased expression of HO-1 is correlated with decreased overall survival in non-small cell lung cancer [43], and it is highly expressed in castration-resistant prostate cancer [44]. There are several potential mechanisms through which HO-1 may affect cancer cell survival. HO-1 can protect cells from proapoptotic factors [45,46] by targeting the p38 subunit of MAPK for proteasomal degradation [47]. It may also reduce the immune response, as HO-1 directly interacts with STAT3 [48]. The role of HO-1 in angiogenesis is well established. HO-1 is a downstream target of stromal cell-derived factor 1 in endothelial cells, and it promotes angiogenesis in a VEGF-independent fashion. In a mouse xenograft model, not only did HO-1 inhibition decrease microvessel density, but it also decreased VEGF and HIF-1α levels [49]. High HO-1 has also been associated with increased microvessel density in bladder tumors [50]. HO-1 may also activate extrinsic mechanisms of metastasis in the tumor microenvironment, as the expression of HO-1 was found to be increased in metastatic prostate tumors, and HO-1 overexpression in macrophages led to increased expression of EMT markers and promoted tumor growth [51].

A growing body of evidence indicates that dysregulation of Nrf2-ARE signaling is a common mechanism of resistance to chemotherapy [52], and recent studies in this area have provided insight into the molecular mechanisms responsible for this phenomenon. For example, Nrf2 expression was increased in cisplatin-resistant bladder cancer cells, and knockdown of Nrf2 was sufficient to partially restore cisplatin sensitivity [53]. In this case, Nrf2-mediated resistance to cisplatin may be due to increased expression of ATP-binding cassette subfamily F member 2 (ABCF2), an Nrf2 target gene that encodes a transmembrane transport module that promotes drug efflux [54]. Elevated ABCF2 levels were found in cisplatin-resistant ovarian cancers, and upregulation of this drug transporter was directly correlated with Nrf2 expression [55]. In addition, other ABC family members appear to be direct Nrf2 target genes, indicating that this is likely a general mechanism by which Nrf2 promotes resistance to chemotherapy [56]. Nrf2 has also been implicated in acquired resistance to 5-fluorouracil (5-FU). Nrf2 levels were higher in tissues from patients with gastric tumors that had become resistant to 5-FU treatment compared to patients that responded to treatment [16]. Nrf2 levels were also higher in a 5-FU resistant colon cancer cell line, and this was because of active demethylation of the *NFE2L2* promoter by the ten-eleven translocation (TET) family of proteins that play a role in cytosine 5-hydroxymethylation [57]. A similar report demonstrated higher Nrf2 levels in cisplatin-resistant colon cancer cells compared to parental cells that were sensitive to cisplatin. In this case, TET1 was involved in the demethylation of the *NFE2L2* promoter, but the promoter also had increased levels of H3K4 trimethylation, an activating histone methyl mark [31]. Providing another mechanism for Nrf2-mediated resistance to therapy, Del Vecchio et al. reported constitutive nuclear localization of Nrf2 in Twist-dedifferentiated cells, decreasing ROS and increasing drug efflux. However, as opposed to KEAP1 inactivation, they determined that Nrf2 was activated by increased eukaryotic translation initiation factor 2 alpha kinase (PERK) signaling to promote EMT. As a result, pretreatment with a PERK inhibitor sensitized these cells to doxorubicin and paclitaxel [58].

To date, much of the research regarding the regulation of Nrf2 activity has focused on Nrf2 protein levels and its canonical regulation by KEAP1 and proteasomal degradation. However, as KEAP1 is often inactivated or silenced in tumors [22], other mechanisms regulating Nrf2 levels and cellular localization may also play a critical role in disease. Several studies have shown that Nrf2 is the target of multiple kinases, some of which appear to control cellular localization. Apopa et al. found that Nrf2 is phosphorylated on serine 40 by casein kinase 2, and that this phosphorylation event caused preferential accumulation of Nrf2 in the nucleus [59]. This site has also been purported to be phosphorylated by protein kinase C, which caused dissociation from KEAP1 [60]. In contrast, glycogen synthase kinase 3β (GSK3β) and Fyn kinase negatively regulate Nrf2 by increasing its nuclear export [61]. Fyn, acting downstream of GSK3β, phosphorylates Nrf2 on tyrosine 568, and the mutation of this residue to alanine renders Nrf2 export-deficient. Nrf2 is also a substrate of AMP-activated protein kinase (AMPK), which phosphorylates Nrf2 on serine 550, blocking nuclear export. As this residue is found within the nuclear export signal of Nrf2, this phosphorylation event may serve to directly block interaction with export proteins. Phosphorylation by AMPK also enhances Nrf2 stability, although it remains unclear whether this is because of the resultant nuclear localization or an additional mechanism [62]. In addition to phosphorylation, several groups have found that Nrf2 can be acetylated in the nucleus. This modification does not affect Nrf2 localization, but it does block Nrf2 transactivation, adding another layer of post-translational control [21,63].

Recent work has established autophagy as a key regulator of Nrf2 protein levels. Although the ability of autophagy to control Nrf2 levels is KEAP1-dependent, cysteine 151, the main redox-responsive residue in KEAP1, is not required for this process [64]. Sequestosome 1 (SQSTM1; hereafter p62) directly interacts with KEAP1, and this interaction is sufficient to increase Nrf2 activity and half-life [64,65], although the interaction of p62 with KEAP1 is not sufficient to completely abrogate the KEAP1–Nrf2 interaction [66]. Interaction with p62 localized KEAP1 to autophagosomes, promoting its autophagic degradation [65,67,68,69]. As a result, KEAP1 no longer interacts with Nrf2, decreasing the ubiquitination and subsequent proteasomal degradation of Nrf2. One group reported that stabilization of Nrf2 through this pathway plays a role in radioresistance [70]. Thus, Nrf2 activation can be a driving factor in cancer progression and therapeutic resistance, making it a promising target for therapy.

## 3. Targeting Nrf2 Signaling

Activating Nrf2 signaling was long thought to be the Holy Grail for anti-aging, cancer, and neurodegenerative diseases. As such, many compounds that inhibit KEAP1 or activate Nrf2 have been discovered, including natural compounds such as curcumin and resveratrol [71,72,73]. However, considering increasing evidence demonstrating that Nrf2 activation can exacerbate cancer progression and worsen patient prognosis, discovering new molecules that target Nrf2 is becoming an area of intense research. An overview of Nrf2 inhibitors is shown in Table 2.

As KEAP1 is the canonical regulator of Nrf2 expression, it is natural that some inhibitors of Nrf2–ARE signaling utilize KEAP1 as a mechanism of action. One such inhibitor is cryptotanshinone, a natural product from the root of the *Salvia* plant. Cryptotanshinone decreases the levels of Nrf2 by increasing the levels of KEAP1 in the cell [74]. This molecule is of particular interest because related molecules, tanshinone I and dihydrotranshinone, act as Nrf2 inducers, suggesting that this class of molecules may be responsible for tightly regulating cellular ROS levels [98,99]. Another KEAP1-dependent inhibitor is the flavonoid 3′,4′,5′,5,7-pentamethoxyflavone, which also acts to increase levels of KEAP1 protein [75]. As with the tanshinones, many flavonoids also act as inducers of Nrf2–ARE signaling [100], although there are other flavonoids that act as Nrf2 inhibitors through various mechanisms (Table 1) [76,92,94,95].

Because KEAP1 and Nrf2 are mutated in many cancer types, particularly in chemotherapy-resistant tumors, targeting pathways that do not rely upon a wild type KEAP1 to suppress Nrf2 is necessary for successful drug development. As such, several studies have sought to identify novel Nrf2 inhibitors by performing drug screens in cell lines lacking functional KEAP1, employing an ARE-luciferase reporter to screen for compounds that block Nrf2 transcriptional activity. One such compound, halofuginone, decreases Nrf2 protein levels by inhibiting pro-tRNA synthetase, which blocks global protein translation. This compound was discovered using A549 lung cancer cells, which harbor constitutively active Nrf2 due to an inactivating mutation in KEAP1. Halofuginone selectively targeted Nrf2-addicted cancer cells, sensitizing them to chemotherapy [93]. Combined treatment of halofuginone and cisplatin also decreased tumor growth in a mouse xenograft model. Another recently identified Nrf2 inhibitor is the ARE expression modulator 1 (AEM1). This inhibitor was discovered in a screen utilizing MYC-3T3-ARE-LUC cells, which overexpress the oncogene c-Myc to drive Nrf2 levels past the suppressive ability of KEAP1. AEM1 decreased Nrf2 target gene transcription and acted synergistically with ROS-producing chemotherapy, although AEM1 treatment alone reduced tumor growth in vivo compared to control-treated mice [85]. AEM1 did not reduce Nrf2 levels, suggesting a KEAP1-independent mechanism. The latest molecule to come out of this type of screen is ML385 [96]. The authors used A549 cells to identify KEAP1-independent small molecule inhibitors of Nrf2. As with AEM1, treatment of cancer cells with ML385 decreased Nrf2 target gene transcription and sensitized to chemotherapies both in vivo and in vitro [96].

While high-throughput drug screening efforts to identify Nrf2 inhibitors have produced promising hit compounds, determining a mechanism of action for these compounds has proven challenging. Several investigators have taken a more rational approach, examining KEAP1-independent mechanisms of Nrf2 regulation and testing inhibitors that affect these pathways. For example, the natural compound trigonelline inhibits Nrf2 activity by blocking its nuclear import [81]. The trigonelline-induced reduction of Nrf2 signaling blocks proteasome activity, sensitizing tumor cells to apoptosis in vitro and reducing tumor growth in vivo when combined with etoposide [82]. The mechanism of action for this compound is not only KEAP1-independent, but also appears to be unrelated to the antioxidant function of Nrf2. Mercado et al. recently discovered that acetylation of Nrf2 decreases its transactivation capability. As a result, treatment with trichostatin A, a histone deacetylase inhibitor, reduced Nrf2 activity in a dose-dependent manner, albeit only reaching a maximum of 53% inhibition [63]. Wang et al. first discovered that treatment with all*-trans* retinoic acid significantly reduced Nrf2 activation without affecting its nuclear localization [87]. Additional studies determined that direct interaction of the retinoid X receptor with Nrf2 was responsible for this observed inhibition of Nrf2 activity [101]. Our group recently identified the proviral integration site for Moloney murine leukemia virus (PIM) kinases as a novel oncogenic signaling pathway that controls Nrf2 activation. We observed that a pan-PIM kinase inhibitor, AZD1208, was preferentially toxic toward hypoxic cancer cells. Mechanistically, PIM inhibition blocked the nuclear localization of Nrf2 in response to hypoxia, allowing cellular ROS to accumulate to toxic levels [88]. The universal increase in ROS associated with hypoxic cells and within solid tumors provides the rationale and highlights the need for the further identification and development of Nrf2 inhibitors as anticancer therapies. Taken together, these studies underscore the importance of examining and understanding the molecular pathways controlling Nrf2 in cancer cells and within the tumor microenvironment.

## 4. Hypoxia and the Oxidative Stress Response

Hypoxia is defined as a physiological state of low oxygen in the cellular environment. Hypoxia occurs in various disease processes, such as ischemia and myocardial infarction, as well as during normal physiological processes, such as exercise and during the adjustment to increased altitude [102]. However, this process is of particularly interest in solid tumor biology. Because the upper limit of oxygen diffusion is ~200 μm, as a tumor proliferates, it rapidly outgrows its existing vasculature (and source of oxygen), which creates hypoxic regions throughout the tumor [103]. To overcome this microenvironmental stress, tumor cells adapt by turning off oxidative phosphorylation, increasing glycolysis, and promoting angiogenesis [104,105]. The main regulator of the cellular hypoxic response is the HIF-1 transcription factor. HIF-1 is composed of an oxygen-dependent subunit (HIF-1/2/3α) and a constitutively expressed subunit (HIF-1β). In the presence of normal physiological levels of oxygen, the HIF-α subunits are hydroxylated on specific proline residues within the oxygen-dependent degradation domain by prolyl hydroxylase domain containing proteins (PHDs). The hydroxylated form of HIF-α is then recognized by the von Hippel Lindau protein, which targets HIF-1/2/3α for ubiquitination and degradation by the 26S proteasome. However, in the presence of low oxygen, PHDs are inactive and HIF-α is no longer degraded, allowing it to accumulate and translocate into the nucleus where it activates genes containing a hypoxia response element [7]. Many HIF-1 target genes are pro-angiogenic factors, such as VEGF and angiopoietin [105]. These factors stimulate the growth of neovessels, rescuing tumor cells from their hypoxic state and promoting tumor progression. In addition, the expression of a hypoxic gene signature has been demonstrated to correlate with increased metastasis, changes in cellular metabolism, and chemoresistance [106].

Mitochondria are the major source of cellular oxygen consumption. However, there are several limitations to the mitochondria acting as the sensor that relays information regarding oxygen levels throughout the cell, as outlined in a recent review [107]. In recent years, it has become evident that ROS also serve as a signaling mechanism for low levels of cellular oxygen. Mitochondria can generate superoxide radical anions at Complexes I, II, and III; cytochrome c, the electron acceptor from Complex III, is considered to play the most prominent role in the cellular response to hypoxia [108]. It is possible that reduced cytochrome c, the form that is unable to accept an electron from Complex III and thus causes Complex III to produce reactive oxygen, is a direct sensor of cellular hypoxia, as this molecule directly donates its electrons to molecular oxygen. When cellular oxygen stores are low, Complex III cannot properly shuttle its electrons and thus creates reactive oxygen species, signaling a hypoxic response [109]. Although it has been suggested that mitochondria are unnecessary for HIF-1 activation [110], other studies have shown that mitochondrial ROS production is required for the cell to mount a hypoxic response [111,112,113].

As the first step in initiating the canonical degradation of HIF-α subunits, PHDs require molecular oxygen as a substrate for HIF-α hydroxylation [102]. These factors have a relatively high Km for oxygen, indicating that they are more sensitive to oxygen deprivation other than hydroxylases [114]. Thus, oxygen sensing is likely the strongest factor influencing PHD activity. However, PHDs also require Fe(II) and 2-oxoglutarate as cofactors [115]. Both of these cofactors are sensitive to the redox state of the cell and may be regulated by ROS production. PHDs are particularly sensitive to the redox state of iron. An in vitro study showed that, in conditions lacking iron or in the presence of Fe(III) chelators, PHDs were unable to hydroxylate the HIF-1/2α subunit. This is also true when cells are depleted of ascorbate, the necessary cofactor for the reduction of Fe(III) to Fe(II) [116]. The protein levels of HIF-α isoforms were decreased in cells upon the addition of ascorbate, and this effect was abrogated by the addition of the PHD inhibitor dimethyloxalylglycine [117]. In addition, the accumulation of ROS has been proposed to negatively impact PHD function. It is hypothesized that PHDs dimerize through the oxidation of cysteine residues in the presence of peroxide, which significantly decreases their hydroxylation activity [118].

Reactive oxygen species can also promote HIF-1 signaling through non-PHD-related mechanisms. Factor inhibiting HIF-1 (FIH-1) hydroxylates the HIF-1α subunit on arginine 803, which decreases the transactivation ability of HIF-1 [119,120]. Though this reaction also requires oxygen, FIH-1 is less sensitive to oxygen deprivation than are the PHDs [121]. However, this hydroxylation reaction is more sensitive to the presence of peroxide, and can thus be more easily inhibited by ROS [122]. Additionally, increased ROS promote the nuclear localization of NF-κB, which increases the transcription of *HIF1A* and its downstream targets [123]. Thus, there is considerable crosstalk between key signaling molecules involved in oxidative stress and hypoxia.

Controlling inflammation is critical to preventing and treating various diseases, including cancer. HIF-1 has been described as a key regulator of the inflammatory response and immune cell function [124]. Although it is well established that hypoxia activates a pro-angiogenic response to recruit endothelial cells to tumors, it can also recruit fibroblasts and tumor-associated macrophages (TAMs) [125]. This can be done both through factors secreted by tumor cells and chemical changes to the microenvironment, such as changes in pH [126]. HIF-1α can also directly augment the recruitment of CD45+ cells and promote TAM polarization [127,128]. Cancer-associated fibroblasts contribute to tumor growth and metastasis through effects such as remodeling of the extracellular matrix [129], and ROS may be involved in their differentiation [130]. Nrf2 also affects inflammation through indirect and direct mechanisms. First, Nrf2 indirectly blocks inflammation by enhancing the expression of antioxidant genes, reducing intracellular levels of ROS. Second, recent work demonstrates that Nrf2 directly blocks the transcription of pro-inflammatory cytokines, including IL-6 and IL-1β [131].

Hypoxia also plays a key role during mammalian development, as well as in the maintenance and differentiation of stem cells in adult tissues. During the later periods of gestation, the rate of vascular formation is insufficient to keep up with the rapid growth of the embryo, and HIF-1 signaling is essential for the survival of embryonic tissues that undergo intermittent periods of hypoxia [132]. HIF-1 has also been shown to play a role in developmental morphogenesis [132]. Many types of adult stem cells reside in hypoxic niches [133], and it is believed that the hypoxic environment allows the stem cell to maintain pluripotency and, when necessary, expand the stem cell population [134,135]. Changes in oxygen concentration also impact stem cell differentiation [136]. This process must be carefully regulated, as cells must migrate from the niche and undergo several rounds of amplification prior to terminally differentiating. Cells that fail to properly differentiate can retain stem-like properties and may become cancer stem cells, which are thought to be more metastatic and have greater proliferative potential [137].

Hypoxia and increased HIF-1 signaling are significantly correlated with chemoresistance in various types of solid tumors. One means by which the cellular response to hypoxia promotes survival is by increasing the expression of antiapoptotic factors and decreasing the expression of proapoptotic factors. In colon tumors, immunohistochemical analysis demonstrated that regions of tumor that stained positive for pimonidazole, a hypoxia probe, displayed decreased levels of the proapoptotic Bcl family members, BH3-interacting domain death agonist (BID) and Bcl2-associated X (BAX). This observation was recapitulated in colon cancer cells, as decreased expression of both BID and BAX was correlated with the degree of oxygen deprivation. BID may be downregulated through direct repression by HIF-1, as HIF-1 bound the *BID* promoter in vitro and HIF-1 knockdown abrogated BID downregulation, but not BAX downregulation, in hypoxia. It is likely that this direct repression plays a role in hypoxia-mediated resistance to etoposide [138]. Hypoxia also downregulated proapoptotic factors in leukemia cells, although this may be a general protective effect, as this was observed in the absence of chemotherapeutic drugs [139]. Hypoxia can also increase the expression of pro-survival genes in a HIF-1-independent fashion. PIM1 has been described as a critical regulator of tumor cell survival in hypoxia. In response to hypoxia, PIM1 and PIM2 are upregulated at the protein level, independent of HIF-1-mediated transcription [76]. One potential mechanism for the upregulation of PIM1 in hypoxia is stabilization of PIM1 mRNA. Blanco et al. showed that hypoxia stabilizes PIM1 mRNA through interaction with the mRNA-binding protein ELAV-like 1, and siRNA-mediated knockdown of PIM1 increased sensitivity to oxaliplatin in hypoxic cells only, suggesting a method for specifically targeting hypoxic regions in vivo [140]. PIM1 has been shown to play a role in hypoxia-induced cisplatin resistance in pancreatic cancer cells, and a kinase dead form of PIM1 was sufficient to resensitize the cells to cisplatin, even in hypoxia [141]. Another factor suggested to promote survival in hypoxia is lysine demethylase 3A (KDM3A), a jumonji-domain-containing histone demethylase that removes methylation at histone H3K9. Like the PHDs and FIH-1, KDM3A requires oxygen and 2-oxoglutarate, but its Km for oxygen is sufficiently low that KDM3A is active even in severe hypoxia (0.5% O_2_). KDM3A mRNA is induced in hypoxia, and KDM3A is recruited to the promoter of the prostate-specific antigen gene by HIF-1 [142]. Thus, KDM3A could represent a mechanism of resistance to androgen deprivation therapy, a major problem in the treatment of prostate cancer.

Among the HIF-1 target genes are a family of glucose transporters [143,144]. It is well established that there is a metabolic shift from the tricarboxylic acid cycle to glycolysis in hypoxia. Thus, it is not unexpected that hypoxia can also promote chemoresistance through changes in cellular metabolism. Doxorubicin-resistant acute myeloid leukemia cells display higher levels of HIF-1α and increased glucose consumption, and blocking glycolysis in these cells resensitizes them to doxorubicin [145]. Moreover, higher levels of HIF-1α and glucose transporters were observed in lung cancer patient tissues following chemoradiotherapy, and heightened expression of HIF-1α, glucose transporter 1, and carbonic anhydrase IX was significantly associated with worse overall and disease-free survival [146].

Another important mechanism by which hypoxia causes chemoresistance is by increasing the expression of transporters, particularly the multidrug resistance protein (MDR-1). The expression of HIF-1 and MDR-1 is significantly correlated in colorectal and gastric cancer tissues [147,148,149]. Moreover, bladder cancer tissues resistant to cisplatin treatment had higher levels of both of these proteins, and in vitro studies in bladder cancer cell lines demonstrated that increased HIF-1α levels enhanced MDR-1 expression and promoted cisplatin resistance [150]. Because these cells were made resistant to cisplatin through prolonged treatment, HIF-1 induction of MDR-1 may be an intrinsic mechanism by which cancer cells acquire resistance to cisplatin. A similar phenomenon has also been described in pancreatic cancer. In this case, a different ABC family member, ATP-binding cassette subfamily G member 2, was correlated with HIF-1α expression. This family member is a direct target of HIF-1, and promotes resistance to gemcitabine in pancreatic cancer cells [151]. This effect may be mediated by ROS, as NADPH oxidase I (NOX1), a generator of ROS, was found to be upregulated in cisplatin-resistant cell lines. Knockdown and overexpression of NOX1 caused subsequent decreases and increases in MDR-1 expression, respectively, and this effect was mediated through HIF-1 signaling [152]. In addition, a separate study by Syu et al. suggests that Nrf2 is involved in drug transporter-mediated chemoresistance in hypoxia. They determined that MCF7 breast cancer cells grown in hypoxia were more resistant to cisplatin, and that knockdown or inhibition of Nrf2 sensitized these cells to cisplatin [153]. The involvement of Nrf2 was related to its regulation of MDR-1 [154]. Because Nrf2 can also activate other ABC family transporters, it is possible that the HIF-1 and Nrf2 pathways interact to promote resistance to chemotherapy, particularly within the hypoxic tumor microenvironment.

## 5. Crosstalk between HIF-1 and Nrf2 

**Figure 1:**
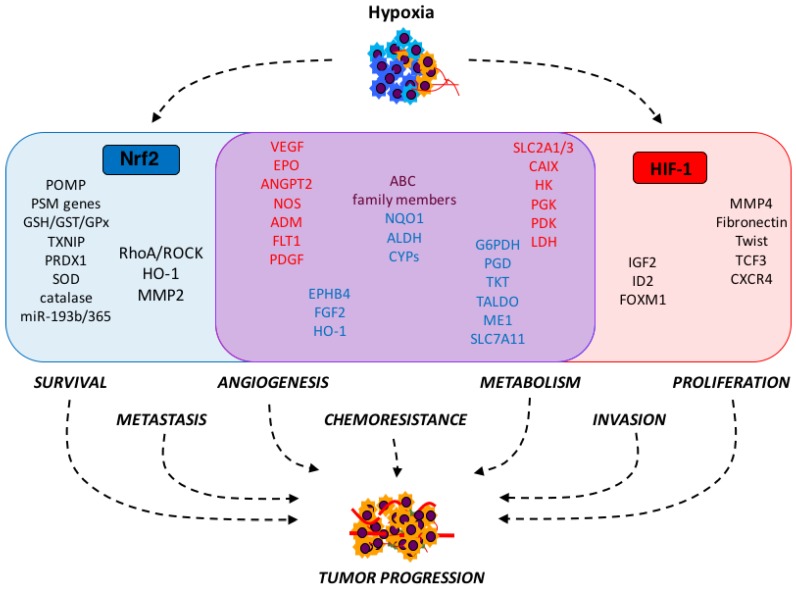
Tumor hypoxia activates Nrf2 and HIF-1 signaling to promote tumor progression through the activation of distinct and overlapping pathways. Through their respective target genes, Nrf2 and HIF-1 activate redundant pathways to stimulate angiogenesis, chemoresistance, and metabolic shifts, as well as unique pathways that contribute to tumor progression, such as survival and metastasis (Nrf2) and invasion and proliferation (HIF-1). Blue box: Nrf2 target genes; red box: HIF-1 target genes. Genes within the purple box (blue text: Nrf2 targets; red text: HIF-1 targets) contribute to redundant pathways. Purple text indicates classes of genes regulated by both Nrf2 and HIF-1. Nrf2: nuclear factor, erythroid 2-like 2; HIF-1: hypoxia inducible factor 1; POMP: proteasome maturation protein; PSM: proteasome; GSH: glutathione; GST: glutathione *S*-transferase; GPx: glutathione peroxidase; TXNIP: thioredoxin interacting protein; PRDX1: peroxiredoxin 1; SOD: superoxide dismutase: miR: microRNA; RhoA: Ras homolog family member A; ROCK: RhoA kinase; HO-1: heme oxygenase 1; MMP: matrix metalloproteinase; VEGF: vascular endothelial growth factor; EPO: erythropoietin; ANGPT2: angiopoietin 2; NOS: nitric oxide synthase; ADM: adrenomedullin; FLT1: Fms-related tyrosine kinase 1; PDGF: platelet-derived growth factor; EPHB4: EPH receptor 4; FGF2: fibroblast growth factor 2; G6PDH: glucose-6-phosphate dehydrogenase; PGD: phosphogluconate dehydrogenase; TKT: transketolase; TALDO: transaldolase; ME1: malic enzyme 1; SLC: solute carrier family; ABC: ATP-binding cassette; NQO1: NADPH: quinone dehydrogenase 1; ALDH: aldehyde dehydrogenase; CYP: cytochrome p450; CAIX: carbonic anhydrase IX; HK: hexokinase; PGK: phosphoglycerate kinase; PDK: pyruvate dehydrogenase kinase; LDH: lactate dehydrogenase; IGF2: insulin-like growth factor 2; ID2: inhibitor of DNA binding 2; FOXM1: forkhead box M1; TCF3: transcription factor 3; CXCR4: C-X-C motif chemokine receptor 4.

Although HIF-1 and Nrf2 signaling are both regulated by the presence of reactive oxygen, there is evidence that these two signaling pathways are not simply linked by cellular context, but that they interact to promote metastasis and play complementary roles in chemoresistance (Figure 1). There is mounting evidence that Nrf2 signaling plays a role in activating and sustaining the HIF-1 response. Several studies have shown that knockdown of Nrf2 is sufficient to decrease HIF-1α at the post-translational level [35,155], suggesting that Nrf2 or its downstream targets play a role in the regulation of PHDs. Lu et al. found that the Nrf2 inhibitor, brusatol, downregulated HIF-1α at the protein level in colon cancer cells by promoting its proteasomal degradation, and this suppressed glucose uptake in these cells [155], suggesting the therapeutic potential of Nrf2 inhibitors in targeting this mechanism of hypoxia-induced chemoresistance. Oh et al. recently discovered that NQO1 expression increases the half-life of HIF-1α protein, and overexpression of NQO1 is sufficient to stabilize HIF-1α levels in normoxia [156]. Mechanistically, NQO1 and HIF-1α physically interact, and overexpression of NQO1 decreases the interaction between HIF-1α and PHDs. The ability of Nrf2 target genes to increase HIF-1 signaling could contribute to the finding that NQO1 is a negative prognosticator for 5-year survival in colorectal cancer [156]. 

Increasing evidence suggests that these stress response pathways can directly and indirectly regulate one another. A recent study attempting to elucidate the sequence of metabolic reprogramming in induced pluripotent stem cells suggests that Nrf2 may be indirectly responsible for the increased transcription of HIF-1α via activation of thioredoxin [157]. Nrf2 has been shown to signal through thioredoxin to increase levels of HIF-1α [158], and HIF-1α can decrease the levels of thioredoxin reductase, thus bolstering the Nrf2 signal [159]. Moreover, HIF-1 signaling and pro-angiogenic factors have been shown to increase Nrf2 activation. Recombinant VEGF can stimulate *NFE2L2* transcription in as little as one hour, leading to elevation of Nrf2 target genes within three hours [160]. However, it is possible that this simply serves as a feed-forward mechanism, as Nrf2 was necessary to promote VEGF secretion in this model. However, these two signaling pathways do not always work in concert or to reinforce each other. Loboda et al. found that, in endothelial cells, HIF-1α stabilization repressed Nrf2 signaling through a Bach1-dependent mechanism [161]. In a separate study, treatment with the natural product andrographolide, an Nrf2 inducer [162], decreased HIF-1α expression. Cells pretreated with andrographolide had increased levels of PHDs, and this was rescued by knockdown of Nrf2, suggesting that this molecule activates Nrf2 signaling to actively block signaling by HIF-1 [163]. Taken together, these studies indicate that the HIF-1 and Nrf2 stress response pathways exist in a complex, interactive signaling network.

Owing to the fact that both HIF-1 and Nrf2 are well established as mechanisms of resistance to anticancer therapies, simultaneously targeting these pathways represents an attractive approach for therapeutic development. One compound that has dual activity toward both HIF-1 and Nrf2 is triptolide, a molecule derived from a Chinese herbal extract. Although this molecule does not have an overt effect when used as a single agent, treatment with triptolide sensitizes cancer cells to chemotherapy-induced apoptosis [83,164]. Nrf2 and HIF-1α protein levels are both reduced in cells receiving triptolide. Prior to determining a mechanism of action for triptolide, this compound was observed to produce cytotoxic effects by increasing ROS [165,166], suggesting that this may be a fruitful area of research. Similarly, PIM kinases positively regulate both Nrf2 and HIF-1 in the cellular response to hypoxia. Published and ongoing work in our lab suggest that inhibition of PIM kinases reduces nuclear localization and activation of Nrf2 in hypoxia, while also reducing HIF-1α protein levels [88].

While simultaneously inhibiting HIF-1 and Nrf2 may be an effective strategy for cancer therapy, there are certain contexts where this approach could be particularly disadvantageous. For example, HIF-1 and Nrf2 are both critical for the cellular response to ischemic injury. During myocardial infarction or stroke, tissue in the heart and brain are deprived of blood flow and are exposed to acute hypoxia. In various models of ischemia/reperfusion, HIF-1 and Nrf2 expressions have been demonstrated to activate a protective response via pleotropic mechanisms. By enhancing the expression of glycolytic genes, HIF-1 activation permits higher rates of ATP production from glucose, which is an oxygen-independent process. The ability of HIF-1 to promote the metabolic shift to glycolysis for ATP production after reperfusion is important, because mitochondrial metabolic enzymes can be inhibited by excessive ROS generated during reperfusion [167]. In addition, HIF-1 increases the production of angiogenic factors, such as VEGF, which is critical for restoring efficient blood supply to the damaged tissue [168]. Acute activation of Nrf2 is also cardioprotective following ischemia reperfusion. Mice treated with hydrogen sulfate or 4-hydroxy-2-nonenal to activate Nrf2 prior to cardiac ischemia reperfusion displayed reduced infarct size in vivo, and pretreatment improved recovery time in Langendorff-perfused mouse hearts [169,170].

The relative contribution of Nrf2 and HIF-1 activity to the adaptive response to hypoxia remains an intriguing question in the field of cancer research. Future studies in this area may be of particular relevance for improving the efficacy of anti-angiogenic drugs in the clinic. By nature, VEGF-targeting agents (e.g., sunitinib and bevacizumab) decrease tumor vasculature and increase hypoxia. Thus, developing new inhibitors to target both the HIF-1 and Nrf2 signaling pathways are needed to effectively oppose hypoxia-mediated therapeutic resistance.

## 6. Conclusions

Hypoxia poses a major problem for the treatment of solid tumors. In response to low oxygen, tumor cells activate a coordinated expression of genes that promote angiogenesis and metastasis, and therapeutic resistance. Growing evidence indicates that Nrf2 and HIF-1 signaling both contribute to the oncogenic phenotypes associated with hypoxia. This review provides an overview of the mechanisms by which these pathways promote chemoresistance and contribute to carcinogenesis. Only recently have we come to appreciate the considerable amount of crosstalk between HIF-1 and Nrf2. Understanding the distinct and overlapping contributions of these signaling pathways is an exciting area of research, particularly in the context of the hypoxic tumor microenvironment. There have been successful attempts to target HIF-1 and Nrf2, and blocking these pathways synchronously has great potential for anti-cancer therapy. In the future, determining the molecular mechanisms by which the HIF-1 and Nrf2 signaling pathways communicate and compensate for each other will undoubtedly provide new targets to exploit oxidative stress in cancer and other disease states.

## Figures and Tables

**Table 1:** Mechanisms of constitutive Nrf2 signaling in tumors.

Mechanism of Activation ^1^	Tumor Types
KEAP1-inactivating mutation	Stomach [22], liver [22], colon [22], prostate [22], lung [22], ovarian [24], breast [25], gallbladder [26]
*KEAP1* silencing	Lung [21]
KEAP1 mRNA degradation	Breast [27]
Nrf2-activating mutation	Esophagus [28], skin [28], lung [28,29], larynx [28], head and neck [29]
*NFE2L2* transcriptional upregulation	Prostate [30], colon [31], pancreas [32]

^1^ For a more in-depth examination of these mechanisms, see the review by Kansanen et al. [33]. Nrf2: nuclear factor, erythroid 2-like 2; KEAP1: Kelch-like erythroid cell-derived protein with cap’n’collar homology-associated protein 1; *NFE2L2*: Nrf2 gene.

**Table 2:** Inhibition of Nrf2 signaling.

Inhibitor	Method of Discovery	Mechanism of Action
Cryptotanshinone [74]	Natural product screen	Increases KEAP1 levels
PMF [75]	Flavonoid	Increases KEAP1 levels
Apigenin [76]	Flavonoid	Dysregulates PI3K/Akt signaling
DPP-23 [77]	Developed chemotherapeutic	Unknown
Ethionamide [78]	Anti-tubercular drug	Unknown
G-quadruplex inhibitor [79]	Sequence analysis	Blocks Nrf2 transcription
JQ1 [80]	Screen/combination treatment ^1^	Unknown
Trichostatin A [63]	Pathway inhibition	Reduces Nrf2 protein stability
Trigonelline [81,82]	Natural product screen	Blocks Nrf2 nuclear import
Triptolide [83]	Developed chemotherapeutic	Decreases Nrf2 protein levels
Valproic acid [84]	Combination treatment ^1^	Inhibits Nrf2 nuclear localization
AEM1 [85]	Screen	Unknown
All-*trans* retinoic acid [86,87]	Combination treatment ^1^ Pathway inhibition	Blocks ARE-binding
AZD1208 [88]	Pathway inhibition	Inhibits Nrf2 nuclear localization
Brusatol [89,90,91]	Screen	Increases Nrf2 ubiquitination Global translation inhibition
Chrysin [92]	Flavonoid	Dysregulates PI3K/Akt signaling
Halofuginone [93]	Screen	Global translation inhibition
Luteolin [94,95]	Flavonoid	Decreases Nrf2 mRNA stability
ML385 [96]	Screen	Unknown
Vorinostat [97]	Combination treatment ^1^	c-Myc downregulation

^1^ Nrf2 inhibition was observed when used in combination with another chemotherapeutic agent. Nrf2: nuclear factor, erythroid 2-like 2; KEAP1: Kelch-like erythroid cell-derived protein with cap’n’collar homology-associated protein 1; PMF: 3′,4′,5′,5,7-pentamethoxyflavone; PI3K: phosphatidylinositol-3-kinase; Akt: protein kinase B; DPP-23: (E)-3-(3,5-dimethoxyphenyl)-1-(2-methoxyphenyl)prop-2-en-1-one; ARE: antioxidant response element; AEM1: ARE expression modulator 1. White: KEAP1-dependent. Light gray: Not tested. Dark gray: KEAP1-independent.
